# The Role of Infant Nutrition in the Prevention of Future Disease

**DOI:** 10.3389/fped.2014.00073

**Published:** 2014-07-21

**Authors:** Yigal Elenberg, Ron Shaoul

**Affiliations:** ^1^Pediatric Gastroenterology and Nutrition Unit, Meyer Children’s Hospital of Haifa, Rambam Medical Center, Haifa, Israel; ^2^Department of Pediatrics, Carmel Medical Center, Faculty of Medicine, Technion – Israel Institute of Technology, Haifa, Israel

**Keywords:** infant, nutrition, preventive medicine, intervention, child, adolescent

## Abstract

There is growing evidence that nutrition is part of the environmental factors affecting the incidence of various diseases. The effect starts in the prenatal life and affects fetal growth and continues in early life and throughout childhood. The effect has been shown on various disease states such as allergic diseases, hyperlipidemia and cardiovascular diseases, obesity, type II diabetes and metabolic syndrome, and immunologic diseases such as celiac disease and type 1 diabetes mellitus. It seems that the recommendations of exclusive breastfeeding until the age of 4 months and subsequently exposure to various solid foods has beneficial effect in terms of allergic, immunologic, and cardiovascular diseases prevention. Will these recommendations change the natural course of these diseases is unknown yet, but there is accumulating evidence that indeed this is the case. In this review, we review the evidence of early nutritional intervention and future disease prevention.

The etiology of many diseases is multifactorial and is influenced by both environmental and genetic factors. One of the most significant environmental factors is the food that we and our children consume. This review will not address food as the trigger for disease, but will concentrate on how various diseases can be prevented through food. Table 1 summarizes the diseases preventable by optimal nutrition in early life.

**Table 1:** **Diseases which may be preventable by optimal nutrition during early childhood**.

Disease	Prevention method
Allergies, atopic dermatitis, asthma	Exposure to a wide variety of foods is recommended between weeks 17 and 26. Consider hydrolyzed formulas for infants who are not breastfed and first degree relatives of patients with atopic diseases
Celiac	Exposure to gluten between weeks 17 and 26 while the infant is still breastfed
Hyperlipidemia, atherosclerosis, and hypertension	Limiting saturated and trans-fat in the diet. Diet rich in fiber and whole grains
Obesity	Exclusive breastfeeding up to 4 months
Type 1 diabetes	Exposure to gluten after 3 months old in infants of parents with type 1 diabetes

## Allergies, Atopic Dermatitis, and Asthma

Food allergy has continued to increase in frequency over the last decade. Avoiding allergens during the early months of life not only proved ineffective in prevention but rather increased the prevalence of allergies to different foods (1). Consequently, early exposure is now actually recommended in order to avoid the development of these allergies.

The implications of modernization – changes in microbiota, industrialized food exposure, air, water, and food pollution, antibiotics, lifestyle habits – are probably some of the explanations for an increased incidence of allergies.

### Cows’ milk protein allergy

To date, there are no clear recommendations for the provision of hydrolyzed cows’ milk formulas to infants with a family history of allergy, mainly those who cannot be breastfed for various reasons, for the prevention of cows’ milk protein (CMP) allergy or any other allergy (eczema, asthma, or allergic rhinitis) (2). Canani et al. (3) prospectively evaluated the effect of different dietary management strategies on the rate of acquisition of tolerance in children with cows’ milk allergy (CMA). Otherwise healthy children (aged 1–12 months) diagnosed with CMA were prospectively evaluated. The study population was divided into five groups based upon the formula feed used for management: (1) extensively hydrolyzed casein formula ([EHCF], *n* = 55); (2) EHCF + *Lactobacillus rhamnosus* GG [LGG], *n* = 71); (3) hydrolyzed rice formula (RHF, *n* = 46); (4) soy formula (*n* = 55); and (5) amino acid based formula (*n* = 33). A food challenge was performed after 12 months to assess acquisition of tolerance. The rate of children acquiring oral tolerance after 12 months was significantly higher (*P* < 0.05) in the groups receiving EHCF (43.6%) or EHCF + LGG (78.9%) compared with the other groups: RHF (32.6%), soy formula (23.6%), and amino acid based formula (18.2%). Katz et al. (4) demonstrated that early exposure to CMP is protective against IgE-mediated CMP allergy. Only 0.05% of the infants who were started on regular CMP formula within the first 14 days versus 1.75% who were started on formula between the ages of 105 and 194 days had IgE-mediated CMA (*P* < 0.001). The odds ratio was 19.3 (95% CI, 6.0–62.1) for development of IgE-mediated CMA among infants with exposure to CMP at the age of 15 days or more (*P* < 0.001). Conversely, the prospective German Infant Nutritional Intervention (GINI) (5) study summarized a 10 year follow up following intervention with various types of hydrolyzed formulas given to high-risk infants. This study showed some degree of prevention of allergic diseases, especially atopic dermatitis, mainly by using extensively hydrolyzed casein formulas and partially hydrolyzed whey formulas. No clear benefit was shown for extensively hydrolyzed whey formulas.

Studies have shown that early exposure to solid foods (under 4 months) increases the incidence of different allergies – 2.5 times for eczema (6, 7) and 5 times for food allergy (6). Therefore, the American Academy of Pediatrics (AAP) Committee on Nutrition in its 2000 position paper recommended avoiding solid food exposure until the age of 6 months and use of hydrolyzed formulas in infants with an atopic family history. The Committee also recommended avoiding common food allergens until a later age – cows’ milk products after 1 year, eggs after the age of 2 years, and fish after 3 years. These recommendations later became widely accepted. In 2001, the World Health Organization (WHO) issued a recommendation for exclusive breastfeeding until the age of 6 months – intending to delay solid food exposure.

After reports of an increasing incidence in various allergies (8), the European Society of Pediatric Gastroenterology, Hepatology and Nutrition (ESPGHAN) Committee of Nutrition recommended in a position paper in 2008 to start exposing infants to solids, including known allergens, between the ages of 17–26 weeks (9). This is supported by the work of Du Toit et al. (10) that showed that the prevalence of peanut allergy (PA) in the UK was 1.85%, and the prevalence in Israel was 0.17% (*P* < 0.001). The adjusted risk ratio for PA in primary school children was 9.8 (95% CI, 3.1–30.5) between countries. Peanut is introduced earlier and is eaten more frequently and in larger quantities in Israel than in the UK.

Further studies published after this position statement have demonstrated that delayed exposure to solid food does not prevent various allergic symptoms (11), but rather contributes to increased incidence of atopic dermatitis, asthma, allergic rhinitis, and allergic tendency, especially with delayed exposure to certain foods such as wheat, oats, cows’ milk, fish, and eggs (12–14). In addition, it was not shown that early exposure causes an increase in the incidence of various allergies.

Moreover, studies showed that later exposure to foods such as potatoes (over 4 months), oats (over 5 months), wheat (over 6 months), meat (over 5.5 months), fish (over 8 months), and eggs (over 10.5 months) was significantly associated with the development of food and inhaled allergies (15).

The reasoning behind the above studies is based on animal studies which demonstrated that tolerance to different foods is a process mediated by early oral exposure. Environmental factors affect the above process and timing and their interaction play a central role: colon colonized by various bacteria, nursing, and exposure to different immunomodulators during the early years of programed training (16).

There is emerging evidence on the development of allergic processes before the age of 4 months, either by proteins transferred through the placenta, breast milk, or dust (17). A recently published review (18) links a diet rich in vitamin D, vitamin E, and Pre- or pro-biotics given to the mother during pregnancy and reduction in the risk of developing asthma later in life. There is growing evidence that administration of probiotics during pregnancy can lower future incidence of atopic diseases in the newborn. This advantageous effect is being debated and probably depends on several factors (strain of bacteria, dose, and host dependent factors) (19).

In summary, early exposure to diverse foods including known allergens is recommended between weeks 17 and 26. Exclusive breastfeeding is recommended until then and continuation of breastfeeding while exposure to a wide variety of foods occurs is highly recommended.

## Celiac Disease

Celiac disease (CD) is a multifactorial autoimmune disorder caused by exposure to gluten in those who have a genetic predisposition for the disease.

A large study conducted in the USA and published in JAMA in 2005 (20) confirmed the hypothesis and found that early exposure (in the first 3 months of life) to gluten significantly increases the risk of developing CD in infants with a genetic predisposition to CD or type 1 diabetes (HLA-DR3/4) or first degree relatives of celiac patients compared with those exposed between ages 4–7 months. Infants exposed after the age of 7 months also showed an increased, but less significant risk of developing CD.

A systematic review (21) published in 2012 of the PREVENT CD group, summarized the research on the prevention of celiac disease as follows:

Breastfeeding and CD – there are studies demonstrating a protective effect of breastfeeding against CD, while some studies have not. The PREVENT CD concluded that there is not enough evidence that exclusive breastfeeding can prevent or delay the onset of CD. They state that most studies support the hypothesis that breastfeeding during the first exposure to gluten is associated with lower incidence of CD. It is unclear whether the effect is prevention or postponement. The PREVENT CD group concludes that according to published studies, it is impossible to link these as cause and effect. In addition, early exposure (below 3 months) or late (7 months and older) demonstrated an increase in the prevalence of CD and probably should therefore be avoided. The amount of gluten exposure during weaning and after may also be related to development of CD.

No interventional studies have examined whether pro- or pre-biotics can affect the development of celiac disease. There are studies that demonstrate flora composition changes in infants with a genetic predisposition to CD.

In conclusion, based on the current data, the ESPGHAN Committee of Nutrition recommended in 2008 avoiding early exposure to gluten before 4 months of age or later than 7 months. The exposure should be gradual, while the baby is breastfed. The AAP recommended in 2012 that exposure to different foods can be started between 4 and 6 months and exposure to gluten is recommended while the infant is on exclusive breastfeeding.

## Heart Disease – Hyperlipidemia, Atherosclerosis, and Hypertension

Nutrition has an effect on a range of cardiovascular diseases (22, 23). This has led to dietary recommendations for adults and children for preventing these diseases. Many studies found a clear link between rapid weight gain in the first months of life and the increased incidence of later cardiovascular diseases (24). Since weight gain on breastfeeding is more subtle, breastfeeding is the preferred way to feed infants.

### Dietary recommendations

Limitation of saturated fat in the diet of up to 7–10% of total daily calories consumed is recommended. Saturated fat is found mostly in meat products and hard fat cheeses, butter, and palm oil. Saturated fat raises LDL cholesterol, and to a lesser extent HDL. Saturated fat increases the risk for atherosclerosis.

Limiting the amount of trans-fat to a minimum. Trans-fat is a vegetable fat changed from “cis” to “trans” structure. It is found in processed foods: microwave popcorn and packed snacks. Foods with a long shelf life usually contain trans-fat. It raises the level of LDL and lowers the level of HDL.

Polyunsaturated fat (PUFA) consumption should account for 10% of total daily calories. Found in vegetable oils – soybean, sunflower, corn and canola, fish oil, nuts, seeds, and mayonnaise, it reduces the level of LDL.

Monounsaturated fat consumption should be 10% of total daily calories. Found in olive oil, canola, and avocado, monounsaturated fat raises HDL.

In general, it is recommended to limit the daily amount of cholesterol in the diet. Adults and children should consume up to 300 mg per day. In children, fats should account for 30–35% of daily calories at the age of 2–3 years and 25–35% between 4–18 years (25).

Whole grains and fiber – whole grains are grains that have been processed and contain the shell, endosperm, and germ, for example, brown rice, whole wheat, and oats. Fibers are polysaccharides that are not digested or absorbed in the small intestine. A diet rich in whole grains and fiber reduces risk for coronary heart disease and cardiovascular mortality, hypertension, diabetes, and obesity.

It is recommended to eat the fruit and vegetable as native foods (rather than juice, for example), preferably using cooking methods that preserve the nutritional components such as evaporation, preferably root vegetables and dark colored fruits (spinach, carrot, peach, and berries).

As a general rule, for adults and children through to adolescence, it is recommended to consume at least five servings of vegetables and fruit (500 g) a day and to incorporate legumes and whole grains. During adolescence, it is recommended to consume between 5 and 8 servings of fruits or vegetables per day (25).

Nuts and almonds improve the level of LDL cholesterol, raise HDL, and lower triglycerides. The recommendation for adults is about 30 g of unsalted nuts per day. Children should consume between 3 and 5 servings of 30 g per week.

## Obesity

Obesity is one of the leading causes for early mortality and disease (26, 27). Prevalence among children is increasing. One of the main reasons is poor nutrition of infants and children, as well as epigenetic effects on the fetus in the context of maternal nutrition during pregnancy.

A balanced diet and physical exercise should lead to a reduction in the increasing incidence of obesity.

Prenatal factors such as low energy intake during the first half of pregnancy increases the prevalence of obesity in children later.

Most studies show that exclusive breastfeeding, probably until the age of 4 months (28), is associated with decreased prevalence of obesity in children. One of the proposed mechanisms is regulation of the amount of food consumed by a breastfed infant, a mechanism that does not exist in bottle fed infants.

The relation between time of exposure to varied foods and obesity is unclear and it is difficult to reach definitive conclusions in this regard.

Various studies have demonstrated that administration of protein-rich formulas (2.6–3 g of protein per kilogram per day) is associated with increased body fat percent by 30% at age 8–10 years. The significant age in this respect is likely to be between 1–2 years. In contrast, increased fat intake (>35% of daily calories) until 2 years of age was not associated with obesity. Fruits and vegetables have a low calorie content and contribute to satiety, thus decreasing caloric intake.

In summary, exclusive breastfeeding until the age of 4 months, is probably, recommended among other things, to prevent obesity in the future.

A diet rich in fruits and vegetables is also recommended later in life and helps maintain appropriate calorie balance.

## Diabetes and Metabolic Syndrome

For over two decades, the link between fetal and post-fetal growth and the development of type 2 diabetes and metabolic syndrome has been recognized – the thrifty phenotype hypothesis (29). According to this theory, the primary cause for type 2 diabetes is environmental; the primary injury is due to malnutrition of the fetus and neonate resulting in poor development of pancreatic beta cells and insulin resistance.

Later obesity (around age 7 years) is another risk factor for the development of metabolic syndrome and type 2 diabetes.

Another effect is epigenetic (30, 31) – the embryonic environment directly affects the methylation of genes, since nutrition is one of the environmental impacts, there is a link between maternal diet during pregnancy and the development of various chronic diseases such as diabetes at the genome level.

Aside from the obvious recommendations mentioned above (heart disease and obesity), no additional nutritional recommendations to prevent type 2 diabetes and metabolic syndrome exist.

For type 1 diabetes, early exposure to gluten (before the age of 3 months) in infants of parents with type 1 diabetes was associated with type 1 diabetes (five times more compared to those exposed after the age of 3 months) (32). This study found no correlation between the duration of breastfeeding and the development of type 1 diabetes. This is in contrast with previous studies that found an association between short duration of breastfeeding and development of type 1 diabetes (33).

In conclusion, there is growing evidence that nutrition is one of the environmental factors affecting the incidence of various diseases. The effect starts in the prenatal life – apparently at the epigenetic level, during pregnancy – maternal nutrition affects fetal growth, during early life, and throughout childhood.

It seems that the recommendations of exclusive breastfeeding until the age of 4 months and subsequent exposure to various solid foods has a beneficial effects in terms of protecting against allergic, immunologic, and cardiovascular diseases. Whether these recommendations change the natural course of these diseases is not yet known, but there is increasing evidence that this may indeed be the case.

## Conflict of Interest Statement

The authors declare that the research was conducted in the absence of any commercial or financial relationships that could be construed as a potential conflict of interest.
